# Psychosocial factors, musculoskeletal symptoms, and presenteeism among labor judges

**DOI:** 10.47626/1679-4435-2022-879

**Published:** 2023-08-08

**Authors:** Sérgio Roberto de Lucca, Fauzi El Kadri Filho, Patrícia Maeda

**Affiliations:** 1 Departamento de Saúde Coletiva, Faculdade de Ciências Médicas, Universidade Estadual de Campinas, Campinas, SP, Brazil; 2 Faculdade de Direito, Universidade de São Paulo, Ribeirão Preto, SP, Brazil

**Keywords:** occupational health, occupational stress, workload, musculoskeletal pain, presenteeism, saúde do trabalhador, estresse ocupacional, carga de trabalho, dor musculoesquelética, presenteísmo

## Abstract

**Introduction:**

Labor judges are subjected to productivity goals associated with a workload that does not take into consideration the complexity of their work.

**Objectives:**

To evaluate the relationship between psychosocial factors, musculoskeletal problems, and presenteeism among labor judges.

**Methods:**

A cross-sectional study was conducted with 151 judges who answered a sociodemographic and occupational characterization questionnaire and the Brazilian versions of the Nordic Musculoskeletal Questionnaire, Health and Safety Executive – Indicator Tool, and Stanford Presenteeism Scale. The results underwent a descriptive analysis and Spearman correlation coefficients were calculated.

**Results:**

The psychosocial dimension of demands presented a higher risk of occupational stress, while role had a lower risk. Musculoskeletal problems in the neck, upper back, shoulders, and lower back were more common and affected almost 70% of the participants. Presenteeism was more affected by the avoiding distractions dimension. Almost all psychosocial dimensions had a significant correlation with musculoskeletal symptoms (p < 0.05), especially demands, which also was correlated with total presenteeism and the avoiding distractions dimension.

**Conclusions:**

The work overload observed among labor judges was related to the occurrence of musculoskeletal problems and to a high prevalence of presenteeism.

## Abstract (pt)

**Introdução:**

Os magistrados trabalhistas estão sujeitos a metas de produtividade associadas a uma carga de trabalho que não contempla a complexidade do seu trabalho.

**Objetivos:**

Avaliar a relação entre fatores psicossociais, problemas osteomusculares e presenteísmo em magistrados trabalhistas.

**Métodos:**

Foi realizado um estudo transversal com 151 magistrados, que responderam a um questionário de caracterização sociodemográfica e ocupacional e às versões brasileiras do Questionário Nórdico de Sintomas Osteomusculares, do Health and Safety Executive – Indicator Tool e do Stanford Presenteeism Scale. Foram utilizadas análises descritivas e teste de correlação de Spearman.

**Resultados:**

A dimensão psicossocial de demandas apresentou um risco mais elevado de estresse ocupacional, enquanto o cargo apresentou um risco mais baixo. Problemas osteomusculares em pescoço, parte superior das costas, ombros e parte inferior das costas foram mais comuns e afetaram quase 70% dos participantes. O presenteísmo foi mais afetado pela dimensão de concentração mantida. Quase todas as dimensões psicossociais apresentaram correlação significativa com os sintomas osteomusculares (p < 0,05), principalmente demandas, que também apresentou correlação com o presenteísmo total e a dimensão de concentração mantida.

**Conclusões:**

A sobrecarga de trabalho observada entre os magistrados trabalhistas foi relacionada à ocorrência de problemas osteomusculares e à elevada prevalência de presenteísmo.

## INTRODUCTION

The Brazilian labor court has undergone a process of informatization of court cases, increased work demands, and stricter control through productivity goals, especially after implementation of the electronic court system (processo judicial eletrônico [PJe]). Even though PJe is able to speed up the course of court cases and enable telework, there are worries about the relationship between an increased work pace and the use of computers, especially when associated with high cognitive demand, mental disorders, and work-related musculoskeletal disorders (WMSDs).^1,2^

The informatization of work tasks can lead to increased workload along with repetitive and monotonous tasks, longer static postures, and less variety of movements, resulting in increased musculoskeletal complaints, especially for the upper limbs and neck.^3,4^ Work that involves a high cognitive demand, especially when associated with pressure for productivity goals and a high workload, is related to more complex occupational activities and may imply a higher risk to physical and mental health.^5^ Work overload, defined as excessive demands to be met in a short period of time, as well as other psychosocial factors, such as reduced autonomy and low social support, have a relevant effect on physical and psychological well-being.^6,7^

Although there is a trend towards the approach of individual factors related to the repercussions of work overload on occupational health, some studies indicate that organizational factors should be considered in the genesis and worsening of these adverse health conditions. The UK Health & Safety Executive (HSE) presents Management Standards including seven work areas that involve the primary sources of occupational stress: demands, control, support by management or colleagues, relationships, role, and communication and change.^8^

Organizational aspects such as an increased computer workload, reduced control over one’s work, low social support, and work overload are related to the occurrence of musculoskeletal problems.^9^

Musculoskeletal problems and mental disorders are frequently related in the workplace; work-related stress mainly seems to be associated with the presence of musculoskeletal pains and aches.^10^

Presenteeism can be defined in different ways, but usually it refers to situations where people continue working while feeling unwell and cannot function at their full capacity, or when an individual works while sick.^11^ The concept of presenteeism used in this study involves the active engagement of an employee at work. It is inclusive and focused on cognitive, emotional, and behavioral engagement during work, which seems particularly suitable for assessing presenteeism among workers in high-level jobs and evaluate work beyond the limits of normal working hours and the formal workplace. It involves the understanding that, when workers are physically present at their jobs while sick, they may experience decreased productivity and below-normal work quality.^12^

Physical problems (including chronic pain) and mental disorders (such as depression and anxiety) are highly prevalent and comorbidity is very common in Brazilian workers, being associated with absenteeism and preseenteism.^13^ Musculoskeletal problems and psychosocial factors, as they are able to affect workers’ physical and mental health, may be related to presenteeism alone or in association.^14,15^

### OBJECTIVES

Considering that the work of judges is evaluated through productivity goals and involves high responsibility given the social impact of their decisions, the aims of this study were to assess psychosocial factors, musculoskeletal problems, and presenteeism in this population, as well as relationships between these variables.

## METHODS

This is an exploratory descriptive study with a quantitative approach of judges of a Regional Labor Court (Tribunal Regional do Trabalho [TRT]).

TRTs comprise the trial and intermediate appellate courts for cases in the Brazilian Labor Court, being responsible for evaluating individual and collective cases derived from work relations, respectively.

### PARTICIPANTS

All 406 judges working during data collection were invited to participate. There were 203 substitute judges, 150 main judges, and 53 appellate judges. The participants who accepted to participate in the study but did not fully complete the questionnaire and data collection instruments were excluded. All judges had worked for at least 1 year by the time data collection took place; job tenure was thus not an exclusion criterium.

### DATA COLLECTION

Data collection was performed between November and December 2018, with self-applied instruments available at the SurveyMonkey platform for online questionnaires and surveys. The judges were invited to participate in the study via e-mail at their institutional addresses with a link for accessing the questionnaires. All participants answered a sociodemographic and occupational questionnaire and the Brazilian versions of the Nordic Musculoskeletal Questionnaire (NMQ) and the Health and Safety Executive – Indicator Tool (HSE-IT). The judges who reported having worked while sick in the last 30 days also answered the Brazilian version of the Stanford Presenteeism Scale (SPS-6).

#### Sociodemographic and occupational characterization questionnaire

We developed a sociodemographic and occupational questionnaire specifically for this study, comprising questions related to age, sex, marital status, children, job tenure, current role, mean weekly workload, and disease occurrence.

#### NMQ

The NMQ was created by Finnish researchers with the aim of standardizing the assessment of musculoskeletal symptoms in an occupational context^16^; it was adapted to Brazilian Portuguese.^17^ The instrument contains questions referring to the presence of symptoms in the neck, shoulders, elbows, wrists and hands, dorsal and cervical spine, lower back, hips, thighs, and buttocks, knees, ankles, and feet in the last 12 months and in the last 7 days. It also assesses the repercussion of these symptoms in the development of work, household, and leisure tasks and in the need for consultations with a health professional due to this condition. NMQ results provide a measure of the frequency of problems in each body region and may also explain the number of body segments each individual complains about.

#### Brazilian version of the HSE-IT

HSE-IT is a 7-dimension, 35-item instrument developed by the HSE, which is the executive entity responsible for preventing psychosocial hazards in the United Kingdom. It allows the identification, at an organizational level, of causes of stress related with the main dimensions indicated by workers.^8^

HSE-IT was validated in the English language^18^ and translated and adapted to Brazilian Portuguese.^19^ The results of psychometric analyses allowed us to consider the existence of an equivalence between measures of the instrument’s seven dimensions in relation to its original and adapted forms. The result, obtained for each dimension separately, ranges from 1 to 5 and the “demands” and “relationships” dimensions were inverted so that, in the final results of all seven dimensions, higher scores were representative of better psychosocial workplace conditions, that is, represented lower risks of occupational stress.^20^

#### Brazilian version of the SPS-6

SPS-6 is an instrument for assessing presenteeism; the original scale was developed in English^12^ and was adapted to Brazilian Portuguese.^21^ This instrument has excellent psychometric characteristics, supporting its use in research on the assessment and improvement of health and productivity status of workers.^12,21^ Each question comprises five items, in a Likert-type scale varying from 1 (completely disagree) to 5 (completely agree). The score obtained by the sum of scores for all six questions can vary from 6 to 30 points. The scale measures the participant’s ability to concentrate and execute work despite a health problem through two factors that include focusing on the work process (avoiding distractions) and work outcomes (completed work). Each one of these two dimensions can present a score ranging from 3 to 15, and their sum provides the total result of the scale.^12^

### DATA ANALYSIS

Descriptive analyses used frequency tables with absolute (n) and percentage (%) values, measures of position (mean, median, minimum and maximum values) and dispersion (standard deviation and interquartile range) for all variables. Quantitative variables were correlated through Spearman’s correlation coefficient. P-values < 0.05 were considered statistically significant, and SPSS version 22.0 was used for the analyses.

### ETHICAL ASPECTS

This study was performed in accordance with Resolution No. 466/12 of the National Health Council and complementary resolutions. The research proposal was authorized by the board of directors at the TRT. It was submitted and approved by the Research Ethics Committee at Universidade Estadual de Campinas (Unicamp) with opinion number 2.989.149/2018. The participants were included and their data were used only after filling the informed consent form presented at the study homepage; without it, access to the data collection instrument was not possible.

## RESULTS

Among the 406 judges invited to this study, 162 accessed the informed consent form and agreed to participate, whereas 3 refused participation. Among the 162 who agreed to participate, 11 did not completely answer the data collection instruments and were excluded from data analysis. Therefore, we analyzed data from 151 judges, which corresponded to a 37.2% response rate.

The sample in this study reflected the role and sex distributions within the TRT. Among the participants, 50% were substitute labor judges, 42% were main judges, and 8% were appellate judges. Men and women participated in this study at similar rates. Almost 80% of the judges were married and most of them (82%) had child ren. The participants worked, on average, 50.3 hours a week and had worked in the Labor Court for 14.3 years (Table 1).

**Table 1:** Descriptive analysis of sociodemographic and occupational characteristics (n = 151), state of São Paulo, 2018

Characteristics	n (%)	Mean (SD)	Median (IQR)	Variation
Age (years)		45.85 (8.37)	46.00 (13.00)	3000-69.00
Sex				
Female	76 (50.3)			
Male	75 (49.7)			
Marital status				
Single	11 (7.3)			
Married	119 (78.8)			
Divorced	15 (9.9)			
Separated	4 (2.7)			
Widowed	2 (1.3)			
Number of kids				
0	27 (17.9)			
1	47 (31.1)			
2	59 (39.1)			
More than 2	18 (11.9)			
Role				
Substitute judge	76 (50.3)			
Main judge	63 (41.7)			
Appellate judge	12 (8.0)			
Job tenure at the TRT (years)		14.30 (8.29)	14.00 (16.00)	1.00-33.00
Weekly workload (hours)		50.27 (8.99)	50.00 (11.00)	27.50-80.00

IQR = interquartile range; TRT = Regional Labor Court (Tribunal Regional do Trabalho).

In the last 12 months, participants presented, on average, problems in almost half of the nine studied body regions and in slightly less than 2 body regions in the last 7 days. The psychosocial dimension of demands presented a higher risk of occupational stress (2.30) and role presented a better result (4.28), whereas the other dimensions presented intermediate results. Presenteeism was surveyed only among judges who reported having worked with health problems in the last 30 days (107 participants), and it was more strongly affected by the avoiding distractions dimension, which presented a worse result when compared with the completed work dimension (Table 2).

**Table 2:** Descriptive analysis of the occurrence of musculoskeletal problems, psychosocial factors, and presenteeism (n = 151), state of São Paulo, 2018

Characteristics	Mean (SD)	Median (IQR)	Variation
NMQ			
Problems in the last 12 months	4.37 (2.13)	4.00 (3.00)	0.00-9.00
Inability in the last 12 months	1.32 (1.92)	0.00 (2.00)	0.00-9.00
Consultations in the last 12 months	2.01 (2.21)	1.00 (3.00)	0.00-9.00
Problems in the last 7 days	1.91 (1.89)	1.00 (3.00)	0.00-8.00
HSE-IT			
Demands	2.30 (0.78)	2.20 (1.00)	1.00-4.50
Control	3.08 (0.81)	3.00 (1.20)	1.00-5.00
Institutional support	3.11 (0.74)	3.20 (1.00)	1.20-5.00
Support by colleagues	3.09 (0.81)	3.00 (1.00)	1.00-5.00
Relationship	3.66 (0.74)	3.80 (0.80)	1.00-5.00
Role	4.28 (0.61)	4.40 (0.80)	1.60-5.00
Communication and changes	2.97 (0.80)	3.00 (1.00)	1.00-5.00
SPS-6*			
Total	19.51 (5.41)	19.00 (7.00)	6.00-30.00
Avoiding distractions	7.71 (3.45)	7.00 (6.00)	3.00-15.00
Completed work	11.80 (3.10)	13.00 (4.00)	3.00-15.00

* n = 107.

HSE-IT = Health and Safety Executive – Indicator Tool; IQR = interquartile range; NMQ = Nordic Musculoskeletal Questionnaire; SPS-6 = Stanford Presenteeism Scale.

The occurrence of musculoskeletal problems in the last 12 months and in the last 7 days was higher in the neck, upper back, shoulders, and lower back areas, affecting almost 70% of the participants. Lower back problems were reported more frequently as being responsible for inability to perform daily tasks, and cervical problems were reported as responsible for consultations with health professionals (Table 3).

**Table 3:** Distribution of musculoskeletal problems per body region (n = 151), state of São Paulo, 2018

	Problems in the last 12 months n(%)	Inability in the last 12 months n(%)	Consultations in the last 12 months n(%)	Problems in the last 7 days n(%)
Neck	105 (69.5)	29 (19.2)	56 (37.1)	57 (37.7)
Shoulders	100 (66.2)	31 (20.5)	50 (33.1)	41 (27.2)
Upper back	103 (68.2)	24 (15.9)	52 (34.4)	39 (25.8)
Elbows	42 (27.8)	10 (6.6)	15 (9.9)	14 (9.3)
Wrists/hands	80 (53.0)	19 (12.6)	28 (18.5)	30 (19.9)
Lower back	100 (66.2)	36 (23.8)	45 (29.8)	44 (29.1)
Hips/thighs	38 (25.2)	17 (11.3)	19 (12.6)	21 (13.9)
Knees	53 (35.1)	18 (11.9)	20 (13.2)	22 (14.6)
Ankles/feet	39 (25.8)	15 (9.9)	18 (11.9)	20 (13.2)

Among the participants who reported having worked in the last 30 days with health issues, 56% had a higher ability to concentrate and accomplish work (SPS-6 > 18) whereas 44% had a lower ability to concentrate and accomplish work (SPS-6 ≤ 18) (Table 4).

**Table 4:** Presenteeism among judges (n = 151), state of São Paulo, 2018

Characteristics	n (%)
Health issues in the last 30 days	107(70.9)
SPS-6 > 18: Higher ability to concentrate and accomplish work	60 (39.7)
SPS-6 ≤ 18: Lower ability to concentrate and accomplish work	47 (31.1)

SPS-6 = Stanford Presenteeism Scale.

Almost all psychosocial dimensions, except for institutional support, were significantly correlated with the occurrence of musculoskeletal problems, especially demands and support by colleagues with problems in the last 12 months and demands and role with problems in the last 7 days (Table 5).

**Table 5:** Spearman’s correlation coefficient between psychosocial factors and musculoskeletal problems (n = 151), state of São Paulo, 2018

Characteristics	Problems in the last 12 months	Problems in the last 7 days
Demands	−0.30*	−0.28*
Control	−0.19†	−0.17†
Institutional support	0.07	0.11
Support by colleagues	−0.25‡	0.11
Relationships	0.14	−0.17†
Role	0.13	−0.24‡
Communication and changes	−0.16†	−0.18†

* p < 0.001.

† p < 0.05.

‡ p < 0.01.

Musculoskeletal problems presented a significant correlation with presenteeism. While demands, control, support by colleagues, relationships, and role present a correlation with the avoiding distractions dimension required in presenteeism, role was the only dimension that showed a correlation with completed work (Table 6).

**Table 6:** Spearman’s correlation coefficient of psychosocial factors and musculoskeletal problems with presenteeism (n = 107), state of São Paulo, 2018

Characteristics	Total presenteeism	Avoiding distractions	Completed work
Problems in the last 12 months	−0.14	−0.06	−0.15
Inability in the last 12 months	−0.22*	−0.15	−0.15
Consultations in the last 12 months	−0.21*	−0.14	−0.11
Problems in the last 7 days	−0.22*	−0.12	−0.19
Demands	0.42†	0.47†	0.13
Control	0.24*	0.270.30‡	0.07
Institutional support	0.06	0.04	0.08
Support by colleagues	0.24*	0.250.30‡	0.06
Relationship	0.20*	0.25*	0.06
Role	0.330.30†	0.23*	0.30‡
Communication and changes	0.16	0.15	0.08

* p < 0.05.

† p < 0.001.

‡ p < 0.01.

## DISCUSSION

This study aimed to evaluate the relationship between psychosocial factors, musculoskeletal symptoms, and presenteeism among judges of a federal labor court. Considering that the judges’ productivity is assessed quantitatively (based on the number of decided cases) while their work has high cognitive demands and social relevance, we investigated some aspects of their health in this context.

The result presented by the demands dimension in our study stood out not only in relation to the other instrument dimensions, but also when compared to other studies that used the HSE-IT for assessing the psychosocial factors at work.^6,22^ Only in a study with Italian bank workers the demands score was below 3.0, but even so it was higher than that presented by the judges in our study.^6^ Demands include matters such as workload, standards, and work environment. Since a judge’s work involves high cognitive overload and responsibility and is continuously subjected to productivity goals that do not consider the complexity of each analyzed case, this dimension becomes the main risk of occupational stress among these workers. On the other hand, the high score of the role dimension demonstrated that the judges understand their role within the organization and that the organization guarantees they do not have conflicting roles.

The informatization of work processes has led to a change in work organization that affects the workers’ biomechanical conditions and psychological demands. Increased demand, the pressure of deadlines and goals controlled electronically, the need to maintain concentration for long periods, the sense of urgency, and the tendency to increase control by supervisors are some of the new psychosocial stressors introduced in the workplace. These factors are associated with high levels of muscular effort and tension and insufficient rest breaks and are related to problems mainly in the neck and upper limbs. Just as in other studies with workers who are intense computer users, the participants of our study also presented more complaints of these body regions and of the lower back.^4,5,23^

According to Casaleiro et al.,^24^ the studies that assessed the working conditions of judges in various countries indicate the dissatisfaction of court professionals with their working conditions, especially with work intensity. The judges consider their workload excessive, ever increasing, and emotionally draining, requiring a fast pace and frequent overtime.

This excessive workload is identified as one of the main sources of stress and may have worsened in the last years with court reforms and performance assessment programs for judges, which define productivity standards for professionals and courts, with goals that are established at increasingly higher levels with progressively less human resources.

Psychosocial factors presented important correlations with the occurrence of musculoskeletal symptoms in our study especially work demands both in the last 12 months and the last 7 days. Support by colleagues, control, and communication and changes also presented significant correlations with the occurrence of musculoskeletal symptoms in the last 12 months. Control, relationships, role, and communication and changes presented significant correlations with the occurrence of musculoskeletal symptoms in the last 7 days.

Even though WMSDs are more commonly referred to as work-related injuries in case of biomechanical hazards, these are better understood when considering their multifactorial etiology, which also involves individual characteristics and psychosocial factors at work.^2^ Various studies that analyzed these relationships indicate consistent evidence of an association between psychosocial factors and musculoskeletal problems, especially when cognitive overload outweighs physical overload at work.^25,26,27^

Almost one-third of our participants displayed presenteeism with a lower ability to concentrate and accomplish work, especially due to a low result in the avoiding distractions dimension. In our study, both musculoskeletal problems and psychosocial factors at work were correlated with presenteeism, especially in the demands and role dimensions. Although productivity in some occupations can be assessed by the number of produced items during a certain time period, productivity in jobs that require predominantly cognitive tasks (such as those carried out by judges when analyzing cases and making decisions) is harder to measure. This study used the SPS-6, one of the most widely used measures of presenteeism, with a more functional approach, assessing to which extent health problems could hinder the cognitive, emotional, and behavioral performance of workers.^11^

According to Goto et al.,^28^ workers who do less overtime, have a work environment with adequate support, and have lower work demands and higher control over their work present a significantly lower risk of presenteeism. Just as in our study, both musculoskeletal problems and psychosocial factors were related with presenteeism, especially work demands, which can predict presenteeism mediated by a high level of occupational stress, resulting in health problems.^14,15^ These relationships may suggest that workers will execute their job while sick as a short-term strategy for avoiding decreases in productivity.^29^

In the study by Yi & Kim,^30^ presenteeism was significantly higher among workers who put in more than 40 hours of work per week and among those with high quantitative work demands, low autonomy, high emotional demand, and occupational stress. They suggest that workers involved in a highly demanding work environment tend to feel pressured to come to work even when sick, and they may feel they have no choice but to show up at work even if feeling unwell when they have less authority to make decisions about their work.

Few studies have assessed the health and working conditions of judges, and we were able to demonstrate how work overload may be related to concentration in the development of activities of analyzing and deciding labor cases. Our sample was representative of the studied population, both considering the role and sex distributions. However, our study presents some limitations. Probabilistic sampling would allow a more reliable generalization of our results in relation to the studied population. Our data collection process performed online did not favor the participation of a higher number of judges, resulting in a reduced response rate. Since this is a cross-sectional study, even though important relationships were observed between the studied variables, cause and effect relationships could not be established and future longitudinal studies are required for confirming causal relationships and our findings.

Considering that judges who were on sick leave for health reasons during data collection were not included in this study, illnesses that were not compatible with their work activity may not have been detected, characterizing a healthy worker bias. Another limitation of the study lies in the fact that participants informed both psychosocial factors and the occurrence of musculoskeletal symptoms and presenteeism (all of which are self-reported measures), which may characterize a common source bias.

## CONCLUSIONS

Notably the demands dimension presented a result that represents a high workload among judges and, as well as almost all psychosocial dimensions, presented an important correlation with the occurrence of musculoskeletal problems. The work overload observed among labor judges was related to the high prevalence of presenteeism. Demands were correlated with presenteeism, especially due to their correlation with the avoiding distractions dimension.
